# Competency-Based Medical Education: Are Canadian Pediatric Anesthesiologists Ready?

**DOI:** 10.7759/cureus.22344

**Published:** 2022-02-17

**Authors:** Katherine Bailey, Nicholas C West, Clyde Matava

**Affiliations:** 1 Department of Anesthesia, BC Children's Hospital, Vancouver, CAN; 2 Research Institute, BC Children's Hospital, Vancouver, CAN; 3 Anesthesiology and Pain Medicine, University of Toronto, Toronto, CAN; 4 Anesthesia and Pain Medicine, Hospital for Sick Children, Toronto, CAN

**Keywords:** medical education, entrustable pofessional activities, pediatric anesthesia, professional competence, clinical competence

## Abstract

Background

With the introduction of Competency-Based Medical Education (CBME), the Canadian Pediatric Anesthesia Society (CPAS) surveyed its members to assess their awareness of and prior experience with CBME concepts and evaluation tools, and identify methods for faculty development of CBME teaching strategies for pediatric anesthesia residents and fellows.

Methods

An online survey was sent to CPAS members. Outcomes included respondents’ previous exposure to CBME and the educational support they had received in anticipation of the curriculum. Questions used multi-item Likert scales and a general feedback question.

Results

The response rate was 39% (60/155). Eighty-eight percent of respondents spent ≥50% of their time practicing pediatric anesthesia; 78% and 45% spent at least a quarter of their time teaching residents and fellows respectively. Eighty-three percent were familiar with CBME concepts, and 58% were familiar with Milestones, Competencies, and Entrustable Professional Activities (EPAs). However, 64% had not received any formal training and 52% had not used any CBME evaluation tools. Learning preferences included small group discussions (72%), lectures with questions and answers (Q&A) (62%), seminars (50%), and workshops (50%).

Conclusions

Despite widespread awareness of CBME concepts, there is a need to educate Canadian pediatric anesthesiologists regarding CBME evaluation tools. Faculty development support will increase the utilization of these tools in teaching practice.

## Introduction

There is a shift occurring in medical education from a time-based model to a competency-based model [1,2]. Moving from the traditional pediatric anesthesia fellowship to a competency-based model may be required so as to better align medical education as a continuum of competence. The Royal College of Physicians and Surgeons of Canada is currently introducing Competence by Design (CBD), a multi-year initiative to implement a competency-based medical education (CBME) approach to residency education and specialty practice in Canada and to align Royal College policies and processes with a CBME approach [3].

The training of pediatric anesthesia to Canadian anesthesia residents occurs predominantly within tertiary pediatric hospitals. Anesthesia residents rotate through these pediatric hospitals to acquire training on the safe and effective management of children in the perioperative period. The training of residents is provided by anesthesiologists who are subspecialized in pediatric anesthesia, and who have often completed pediatric anesthesia fellowship training. 

Study aim

The aim of this survey was to elucidate the knowledge and experience of pediatric anesthesiologists with CBME, its major constructs, and its associated evaluation tools in Canadian academic teaching institutions. The secondary purpose is to help identify the resources needed by practitioners to successfully implement competency-based teaching and its evaluation into current pediatric anesthesia academic practice [4-6].

## Materials and methods

Study design and approval

An online survey tool was developed by the authors, using Fluidsurveys (http://fluidsurveys.com), a secure online electronic data capture tool hosted in Canada. The survey was reviewed by the Education Committee of the Canadian Pediatric Anesthesia Society (CPAS), prior to obtaining ethical approval from the University of British Columbia Children’s & Women’s Research Ethics Board (approval number H15-02624). In keeping with previous surveys [7,8], practicing pediatric anesthesiologists were identified as eligible study participants by their membership in CPAS. The majority of CPAS members were working at academic institutions that provide pediatric anesthesia fellowship training. The survey questionnaire was tested beforehand using a convenience sample of five pediatric anesthesia fellows and four pediatric anesthesiologists. 

Study participants

The online survey was distributed to 155 out of 160 CPAS members who had a valid email address in the membership database. The survey remained open for 60 days after the initial email was sent, and a single follow-up reminder email was sent to all eligible study participants two weeks after the initial invitation to participate in the survey. No identifying data was collected and consent was implied by participation. 

Survey instrument

Participants were asked to describe their pediatric anesthesia experience according to four intervals (less than 5 years, 5-10 years, 11-20 years, and greater than 20 years) and their teaching activities according to what proportion of their time (None, <25%, 25-50%, 50-75%, >75%) was spent teaching fellows, residents, and medical students.

Participants were also asked to determine their familiarity with CBME, especially the major concepts of Milestones, Competencies, and Entrustable Professional Activities (EPAs). Participants were asked to rate their experience of these concepts in their own training and in their teaching activities, each according to a 5-point Likert scale (Strongly disagree, Disagree, Neither agree nor disagree, Agree, Strongly agree). 

In addition, participants were then asked about their experience of the following specific CBME evaluation tools, how easy each was to apply in an anesthesia setting, and their accuracy in assessing trainees’ ability: Direct Observation of Procedural Skills (DOPS), Clinical Evaluation Exercise (A-CEX/Mini-CEX), Anesthesia List Management Tool (ANTS/ALMATS), Multi-Source Feedback (MSF), Case-Based Discussions and Simulation.

Finally, participants were asked to indicate their opinion on the value of the CBME approach to pediatric anesthesia training and their preferred formats for acquiring the necessary skills to employ CBME approaches in their teaching (lectures without question and answer sessions, lectures with question and answer sessions, seminars, simulation cases, workshops, journal club, small group discussions, online podcasts, operating room case-based learning, one-on-one consultations with colleagues and/or peer experts and leaders). Respondents were instructed to select as many choices as they felt applicable.

Sample size

Data were collected and managed using Fluidsurveys.com. From a population of 155, a minimum sample of 60 respondents (39%) was required for a confidence level of 95% and a confidence interval (CI) of 10%. For comparison, the average online survey response rate in the medical literature is 18-20% [9]. Respondents were allowed to skip questions.

Data analysis

Data were summarized as proportions of responses in each category using Microsoft-Excel (Microsoft, Redmond, WA). Descriptive statistics (central tendency and distribution) as appropriate for the data distribution and 95% CI for proportions were determined using the Vassar Statistical website (http://vassarstats.net, Vassar College, Poughkeepsie, NY). 

## Results

The online survey was sent to 155 CPAS members. Responses were received from 60/155 members with a survey response rate of 39%. All members worked in hospitals that had not implemented a CBME program during the survey period.

Pediatric anesthesia experience and teaching activities

The median years of practice of respondents were 11-20 years (Table 1). 

**Table 1:** Demographics of survey respondents Total number of participants (N) = 60; n = number of participants

Variable	Response	n	% (95% CI)
Years in practice	Less than 5 years	6	10 (4.7 to 20.2)
	5-10 years	16	27 (17.1 to 39)
	11-20 years	17	28 (18.5 to 40.8)
	More than 20 years	21	35 (24.2 to 47.6)
The proportion of time spent doing pediatric anesthesia	None	0	0 (0 to 6.0)
	<25%	1	2 (0.3 to 8.9)
	25-50%	6	10 (4.7 to 20.2)
	50-75%	9	15 (8.1 to 26.1)
	>75%	44	73 (61 to 82.9)
The proportion of time spent teaching residents	None	1	2 (0.3 to 8.9)
	<25%	12	20 (11.8 to 31.8)
	25-50%	27	45 (33.1 to 57.5)
	50-75%	13	22 (13.1 to 33.6)
	>75%	7	12 (5.8 to 22.2)
The proportion of time spent teaching fellows	None	10	17 (9.3 to 28.0)
	<25%	23	38 (27.1 to 51.0)
	25-50%	20	33 (22.7 to 45.9)
	50-75%	4	7 (2.6 to 15.9)
	>75%	3	5 (1.7 to 13.7)
The proportion of time spent teaching medical students	None	8	13 (6.9 to 24.2)
	<25%	43	72 (59.2 to 81.5)
	25-50%	7	12 (5.8 to 22.2)
	50-75%	2	3 (0.9 to 11.4)
	>75%	0	0 (0 to 6.0)
Administrative positions held currently or previously	None	17	28 (18.5 to 40.8)
	Undergraduate/Medical Student Coordinator	9	15 (8.1 to 26.1)
	Residency Coordinator	12	20 (11.8 to 31. 8)
	Fellowship Coordinator	9	15 (8.1 to 26.1)
	Subspecialty Clinical Director	8	13 (6.9 to 24.2)
	Clinical Director	4	7 (2.6 to 15.9)
	Associate/Assistant Departmental Chief	4	7 (2.6 to 15.9)
	Departmental Chief	5	8 (3.6 to 18.1)
	Department Program Leader	9	15 (8.1 to 26.1)
University appointments held	None	2	3 (0.9 to 11.4)
	Lecturer	3	5 (1.7 to 13.7)
	Assistant Professor	40	67 (54.1 to 77.3)
	Associate Professor	11	18 (10.6 to 30.0)
	Professor	6	10 (4.7 to 20.2)

Almost three-quarters (44/60, 73%) respondents stated that the majority of their time (greater than 75%) was spent practicing pediatric anesthesia, and a further 9/60 (15%) stated that pediatric anesthesia comprised 50-75% of their practice. 

The majority of respondents (47/60, 78%) spent greater than 25% of their time teaching residents. Only one respondent replied as having spent no time teaching anesthesia residents. Less than half of respondents (27/60, 45%) reported spending >25% of their time teaching fellows, with 10/60 (17%) respondents reporting no teaching activities with fellows. The majority of respondents (51/60, 85%) reported spending less than 25% of their time teaching medical students.

Familiarity with CBME concepts and evaluation tools

Fifty out of 60 (83%) respondents replied that they were familiar with the concept of CBME, with a mean response of 4.13 (Agree) (Table 2). The majority (35/60, 58%) respondents replied that they were familiar with the major concepts of Milestones, Competencies, and EPAs. Overall, 38/59 (64%) had not received any formal training in using CBME evaluation tools, and 31/60 (52%) were not currently using nor previously used any CBME evaluation tools. CBD was the most commonly used tool for teaching (used by 49/54, 91%) and evaluation of trainees (used by 40/58 69%) (Table 2). The simulation was found to be actively used by 22/42 (52%) of respondents for teaching, but was incorporated less as an evaluation tool (used by 10/40, 25%).

**Table 2:** Respondents’ familiarity and experience with CBME and various CBME assessment tools CBME = Competency-Based Medical Education Total number of participants (N) = 60 n = Number of participants, data presented as n (%; 95% CI)

	n	Agree/Strongly agree	Neutral	Disagree/Strongly disagree
Familiar with the concept of CBME (vs. time-based medical training)	60	50 (83; 72 to 90.7)	7 (12; 5.8 to 22.2)	3 (5; 1.7 to 13.7)
Familiar with Milestones, Competencies and Entrustable Professional Activities (EPAs)	60	35 (58; 45.7 to 69.9)	15 (25; 15.8 to 37.2)	10 (17; 9.3 to 28.0)
Received formal instruction and training in applying common evaluation tools used in CBME	59	14 (24; 14.7 to 36.0)	7 (12; 5.9 to 22.5)	38 (64; 51.6 to 75.4)
Previously used / currently applying CBME evaluation tools in teaching practice	60	15 (25; 15.8 to 37.2)	14 (23; 14.4 to 35.4)	31 (52; 39.3 to 63.8)
Experience using DOPS (Direct Observation of Procedural Skills)	60	11 (18; 10.6 to 29.9)	6 (10; 4.7 to 20.2)	43 (72; 59.2 to 81.5)
Experience using A-CEX or mini-CEX (Clinical Evaluation Exercise)	59	15 (25; 16.1 to 37.8)	2 (3; 0.9 to 11.5)	42 (71; 58.6 to 81.1)
Experience using ANTS (Anesthesia for Non-Technical Skills) or ALMAT (Anesthesia List Management Tool)	58	7 (12; 6.0 to 22.9)	2 (3; 1.0 to 11.7)	49 (84; 73.1 to 91.6)
Experience using a Multi-Source Feedback (MSF) tool in an anesthesia setting	59	19 (32; 21.7 to 44.9)	5 (8; 3.7 to 18.4)	35 (59; 46.6 to 70.9)
Currently use Case-Based Discussions as part of anesthesia teaching	54	49 (91; 80.1 to 96)	3 (6; 1.9 to 15.1)	2 (4; 1 to 12.5)
Currently use Case-Based Discussions as part of trainee evaluations/feedback	58	40 (69; 56.2 to 79.4)	4 (7; 2.7 to 16.4)	14 (24; 15 to 36.5)
Currently use Simulation as part of anesthesia teaching	42	22 (52; 37.7 to 66.6)	4 (9; 3.8 to 22.1)	16 (38; 25 to 53.2)
Currently use Simulation as part of trainee evaluations/feedback	40	10 (25; 14.2 to 40.2)	8 (20; 10.5 to 34.8)	22 (55; 39.8 to 69.3)
Exposed to a competency-based training and evaluation method during anesthesia training	58	7 (12; 6 to 22.9)	8 (14; 7.2 to 24.9)	43 (74; 61.6 to 83.7)

Respondents experience with specific CBME tools for evaluating competencies in anesthesia 

The majority of respondents did not have any significant experience using DOPS (43/60, 72%), A-CEX/Mini-CEX (42/59, 71%), ANTS/ALMATS (49/58, 84%), or MSF (35/59, 59%) (Table 3). Respondents who had experience using these tools agreed that they found them easy to apply: DOPS (10/22, 45%), A-CEX/Mini-CEX (12/22, 55%), MSF (15/28, 54%), with the exception of ANTS/ALMATS (5/16, 31%). Furthermore, they agreed that these tools were an accurate assessment of a trainee’s abilities: DOPS (9/22, 41%), A-CEX/Mini-CEX (9/23, 39%), again with the exception of ANTS/ALMATS (3/16, 19%). Survey respondents were not specifically asked if they found MSF to be an accurate assessment of a trainee’s abilities. 

Respondents preferred learning modes on the use of competency-based tools

While very few of the respondents were exposed to CBME during their own training (7/58, 12%) (as shown in Table 2), half of the respondents reported that competency-based training and evaluation will be a useful change to implement in pediatric anesthesia fellowship training (Table 3).

**Table 3:** Respondents’ preferred formats for acquiring/enhancing knowledge and use of CBME assessment CBME = Competency-Based Medical Education Total number of participants (N) = 60

Format	n	% (95% CI)
Small group discussions	43	72 (59.2 to 81.5)
Lectures with question and answer sessions	37	62 (49 to 72.9)
Seminars	30	50 (37.8 to 62.3)
Workshops	30	50 (37.8 to 62.3)
Operating room case-based learning	26	43 (31.6 to 55.9)
One-on-one consultations with colleagues and/or peer experts and leaders	25	42 (30.1 to 54.3)
Simulation cases	21	35 (24.2 to 47.6)
Journal club	19	32 (21.3 to 44.2)
Lectures without question and answer sessions	13	22 (13.1 to 33.6)
Online podcasts	13	22 (13.1 to 33.6)

There was a strong learning preference towards small group discussions (43/60, 72%), lectures with question and answer sessions (37/60, 62%), seminars (30/60, 50%), and workshops (30/50, 50%) (Table 4). Interestingly, one of the CBME learning/evaluation tools, simulation cases (21/60, 35%), was not in the top three preferences selected. 

**Table 4:** Respondents’ perception of evaluation tools Total number of participants (N) = 60 n = Number of participants, data presented as n (%; 95% CI)

Tool	Criteria	n	Agree / Strongly agree	Neutral	Disagree / Strongly disagree
DOPS (Direct Observation of Procedural Skills)	Easy to apply	22	10 (45; 26.9 to 65.3)	8 (36; 19.7 to 57.0)	4 (18; 7.3 to 38.5)
	Accurate assessment of trainees’ procedural skills	22	9 (41; 23.3 to 61.3)	9 (41; 23.3 to 61.3)	4 (18; 7.3 to 38.5)
A-CEX or mini-CEX (Clinical Evaluation Exercise)	Easy to apply	22	12 (55; 34.7 to 73.1)	4 (18; 7.3 to 38.5)	6 (27; 13.2 to 48.2)
	Accurate assessment of a trainee’s ability to manage clinically	23	9 (39; 22.2 to 59.2)	10 (43; 25.6 to 63.2)	4 (17; 7 to 37.1)
ANTS (Anesthesia for Non-Technical Skills) or ALMAT (Anesthesia List Management Tool)	Easy to apply	16	5 (31; 14.2 to 55.6)	7 (44; 23.1 to 66.8)	4 (25; 10.2 to 49.5)
	Accurate assessment of trainees’ ability to run an Operating Room list safely and efficiently	16	3 (19; 6.6 to 43)	8 (50; 28 to 72)	5 (31; 14.2 to 55.6)
MSF (Multi-Source Feedback)	Easy to apply in an anesthesia setting	28	15 (54; 35.8 to 70.5)	5 (18; 7.9 to 35.6)	8 (29; 15.3 to 47.1)
Believe competency-based training and evaluation will be a useful change to implement in pediatric anesthesia fellowship training		60	30 (50; 37.7 to 62.3)	22 (37; 25.6 to 49.3)	8 (13; 6.9 to 24.2)

## Discussion

The pending implementation by the Royal College of Physicians and Surgeons of CBME in Canadian anesthesia residency training represents a significant paradigm shift in the teaching and evaluation of pediatric anesthesia. The results of this survey suggest that the Canadian pediatric anesthesia community is familiar with some of the key concepts of CBME, Milestones, Competencies, and EPAs. However, major gaps exist in the knowledge and current use of the full range of CBME evaluation tools for pediatric anesthesia teaching and evaluation of trainees. The faculty has identified small group sessions to be key in gaining information on this new curriculum.

Competency-based pediatric anesthesia training presents opportunities for a meaningful assessment using evaluation tools that are based on a detailed rubric (the milestones), and which are available to both the trainer and trainee. Outcome-based evaluation allows learners to focus on the key concepts and skills to direct their learning. This is in contrast to traditional methods of medical education, which have emphasized a time-based evaluation. Curricula are designed around competency-based milestones that must be clearly defined and agreed upon, including specific milestones for an individual subspecialty, such as has recently been published for pediatric cardiac anesthesiology [10].

Our findings demonstrate that the majority of pediatric anesthesiologists are not familiar with the range of CBME tools and do not have experience with their use. Educating anesthesiologists regarding the best choice of tools to measure specific learning outcomes is vital in order to facilitate constructive feedback to trainees allowing them to improve their learning experience and achieve the required competencies. Educators will need to assist faculty with professional development in the use of DOPS, A-CEX/Mini-CEX, ANTS/ALMATS, MSF, CBD, and Simulation for assessment. Hodges et al. have previously argued that the use of checklist-type tools offers poor discrimination of expertise, but can be used in combination with global rating scales [11]. The new CBME curriculum will also include the assessment of EPAs and milestones. Faculty across Canada will require training on the use of these tools for an effective transition to the CBME model.

Reassuringly for respondents’ familiarity with these evaluation tools, the consensus was that they were easy to apply and perceived to be a reasonable assessment of the milestone, competency, or EPA that they were designed to evaluate. Recent studies [12,13] exist on how to address the primary concerns and challenges of CBME implementation and how to support faculty development in order to “train the trainers”. Boet et al. [14] identified concerns from Canadian Program Directors regarding the administration of CBME, including challenging scheduling and the need to have ‘buy-in’ from faculty. They did not however report on approaches to buy-in or faculty development. A recent study into the implementation and evaluation of an online feedback tool for anesthesia residents, which facilitated mapping of feedback to milestones, demonstrated some perceived benefits for trainees, but also highlighted issues in the uptake of the tool by staff [15].

Our study reports that respondents prefer small group discussions and panel discussions as methods for acquiring training on the CBME process and the use of assessment tools. The inclusion of CBME-focused small group discussions and panel discussions at pediatric anesthesia meetings may assist in addressing this important need during this time of transition. The use of small group sessions will likely be costly. Faculty developers will need to consider the impact of these challenges on delaying the process of implementation and may consider starting with faculty that regularly teach residents. At a single Canadian pilot site, the training of faculty was met with such challenges and these will need to be addressed country-wide with local considerations [16]. Further, the development and validation of pediatric anesthesia EPAs and global rating scales will assist in providing a standardized assessment of fellows within Canada and in other jurisdictions [17].

Study limitations

There are several limitations to our study. This is a survey of pediatric anesthesiologists practicing in Canada and the results may not be translatable to other jurisdictions. However, the training of Pediatric Anesthesia fellows anecdotally is similar across the globe allowing for the consideration of many of the tools and methods we reviewed to be considered in other jurisdictions. Another limitation is the survey is reflecting practice at a point in time. Further surveys or mixed methodology studies may determine other barriers or opportunities for implementing competency-based medical education for Pediatric Anesthesia fellowship training.

## Conclusions

The results of this survey suggest that, despite widespread general awareness of the CBME concept and major constructs, there is a need to educate Canadian pediatric anesthesiologists in the use of specific CBME evaluation tools. Further faculty development is required as one of many factors of many to increase utilization of these tools in teaching practice.
